# Investigation of the Possible Role of Tie2 Pathway and *TEK* Gene in Asthma and Allergic Conjunctivitis

**DOI:** 10.3389/fgene.2020.00128

**Published:** 2020-02-27

**Authors:** Zsófia Gál, András Gézsi, Viktor Molnár, Adrienne Nagy, András Kiss, Monika Sultész, Zsuzsanna Csoma, Lilla Tamási, Gabriella Gálffy, Bálint L. Bálint, Szilárd Póliska, Csaba Szalai

**Affiliations:** ^1^ Department of Genetics, Cell- and Immunobiology, Semmelweis University, Budapest, Hungary; ^2^ MTA-SE Immune-Proteogenomics Extracellular Vesicle Research Group, Semmelweis University, Budapest, Hungary; ^3^ Department of Measurement and Information Systems, Budapest University of Technology and Economics, Budapest, Hungary; ^4^ Institute of Genomic Medicine and Rare Disorders, Semmelweis University, Budapest, Hungary; ^5^ Department of Allergology, Heim Pál Children’s Hospital, Budapest, Hungary; ^6^ Department of Urology, Heim Pál Children’s Hospital, Budapest, Hungary; ^7^ Department of Ear, Nose and Throat, Heim Pál Children’s Hospital, Budapest, Hungary; ^8^ Outpatient Care for Allergy and Asthma, National Korányi Institute of TB and Pulmonology, Budapest, Hungary; ^9^ Department of Pulmonology, Semmelweis University, Budapest, Hungary; ^10^ Adult Inpatient Care, Pulmonology Hospital Törökbálint, Törökbálint, Hungary; ^11^ Department of Biochemistry & Molecular Biology, Genomic Medicine & Bioinformatic Core Facility, University of Debrecen, Debrecen, Hungary; ^12^ Department of Research and Development, Heim Pál Children’s Hospital, Budapest, Hungary

**Keywords:** asthma, conjunctivitis, Tie2 pathway, *TEK* gene, angiopoietin (Ang)

## Abstract

Tie2, coded by the *TEK* gene, is a tyrosine kinase receptor and plays a central role in vascular stability. It was suggested that variations in the *TEK* gene might influence the susceptibility to asthma and allergic conjunctivitis. The aim of this study was to further investigate these suggestions, involving different populations and to study the Tie2 related pathway on a mouse model of asthma. The discovery, stage I cohort involved 306 patients with moderate and severe allergic rhinitis, the stage II study consisted of four cohorts, namely, adult and pediatric asthmatics and corresponding controls. Altogether, there were 1,258 unrelated individuals in these cohorts, out of which 63.9% were children and 36.1% were adults. In stage I, 112 SNPs were screened in the *TEK* gene of the patients in order to search for associations with asthma and allergic conjunctivitis. The top associated SNPs were selected for association studies on the replication cohorts. The rs3824410 SNP was nominally associated with a reduced risk of asthma in the stage I cohort and with severe asthma within the asthmatic population (p=0.009; OR=0.48) in the replication cohort. In the stage I study, 5 SNPs were selected in conjunctivitis. Due to the low number of adult patients with conjunctivitis, only children were involved in stage II. Within the asthmatic children, the rs622232 SNP was associated with conjunctivitis in boys in the dominant model (p=0.004; OR=4.76), while the rs7034505 showed association to conjunctivitis in girls (p=0.012; OR=2.42). In the lung of a mouse model of asthma, expression changes of 10 Tie2 pathway-related genes were evaluated at three points in time. Eighty percent of the selected genes showed significant changes in their expressions at least at one time point during the process, leading from sensitization to allergic airway inflammation. The expressions of both the *Tek* gene and its ligands showed a reduced level at all time points. In conclusion, our results provide additional proof that the Tie2 pathway, the *TEK* gene and its variations might have a role in asthma and allergic conjunctivitis. The gene and its associated pathways can be potential therapeutic targets in both diseases.

## Introduction

Tie2 is a tyrosine-protein kinase receptor located principally on vascular endothelial cells and plays a crucial role in the stability of the vascular system (Zhang et al., 2019). In human there are three known Tie2 ligands, angiopoietin 1 (Ang1), Ang2, and Ang4 with similar affinity for binding to Tie2. The best-studied member, Ang1 stimulates the activity of the kinase by binding to Tie2. The role of Ang2 is rather controversial, it can function as an Ang1 antagonist, although, in some cases, it may act as a partial agonist. Under certain conditions, Ang2 can also promote angiogenesis. Ang4 can modulate Ang1 signaling and also has an antimicrobial effect. Tie2 signaling pathway appears to complement the VEGF pathway by contributing to subsequent stages of vascular development (Brindle et al., 2006; Augustin et al., 2009; Thurston and Daly, 2012).

**Figure 1:**
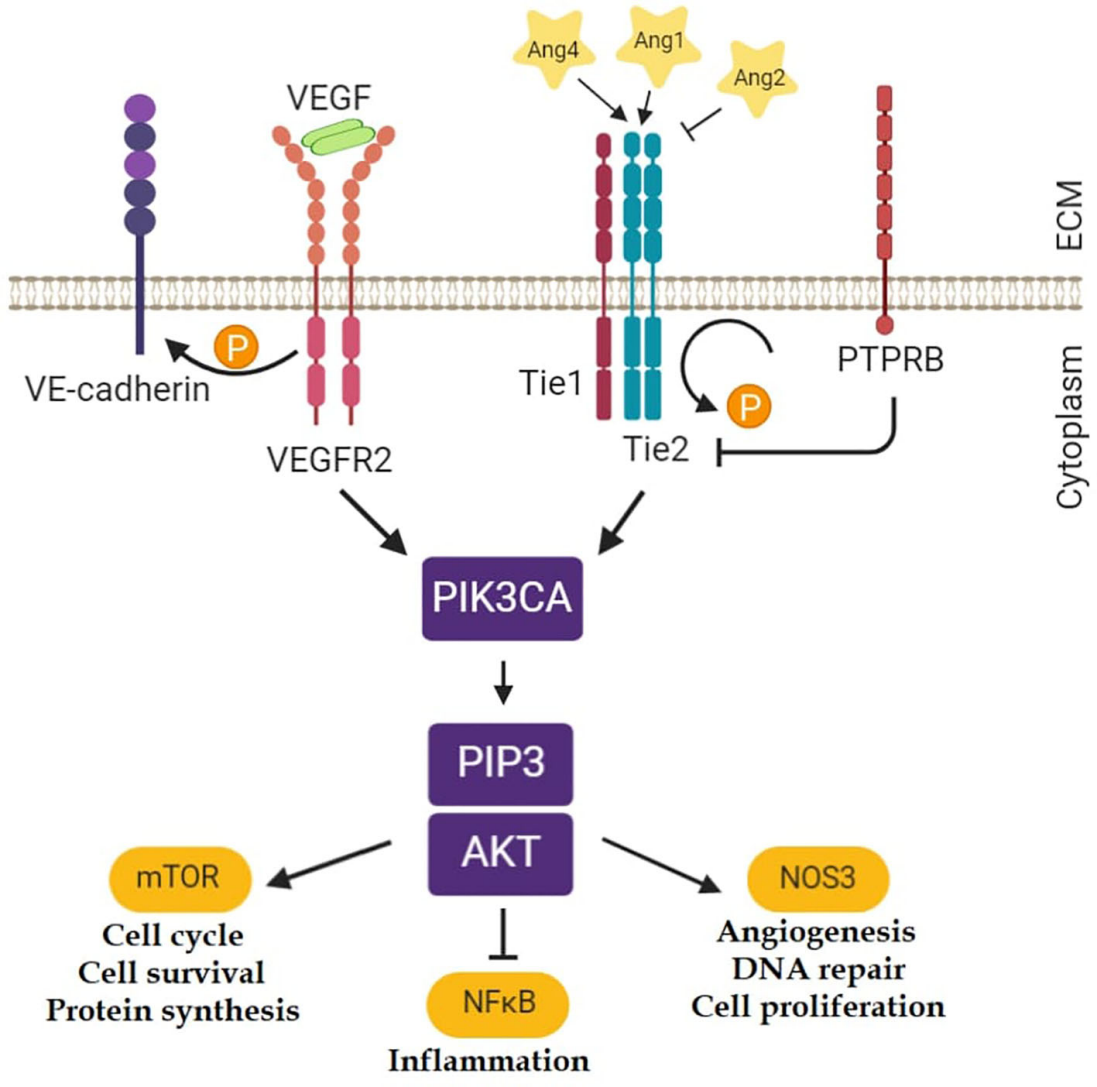
Tie2 related pathways (Karar and Maity, 2011). Ten genes were selected from these pathways for the gene expression analyses in the mouse model of asthma: *Angpt1; Angpt2; Angpt4; Tek; Nfkb1; Kdr* (gene for Vegfr2); *Pik3ca; Akt1; Nos3; Ptprb*. (The figure was created with BioRender.)

Ang1 can support endothelial cell survival through the stimulation of the PI3K-Akt1 pathway, and comparable results could be achieved with elevated concentrations of Ang2. Signaling by the PI3K-Akt1 pathway contributes to eNOS phosphorylation, NO production, and angiogenesis (**Figure 1**) (Karar and Maity, 2011).

The Tie2 receptor is encoded by the *TEK* (TEK Receptor Tyrosine Kinase) gene in humans. *TEK* is abundantly expressed in the lung and in the endothelium of Schlemm’s canal in the eye and the Tie2 pathway has been suggested that it might play a role in asthma and different eye disorders including allergic conjunctivitis (Souma et al., 2016; Fodor et al., 2018).

Asthma is a chronic pulmonary disease affected by a large amount of environmental and genetic factors. Both neovascularization and angiogenesis are also remarkable features of asthma causing structural changes in the airway walls. Increased vascularity and angiogenesis in asthmatic patients contribute to airway occlusion as a result of an increase in airway wall thickness (Temesi et al., 2014; Fodor et al., 2017).

Severe asthma is a subtype of difficult-to-treat asthma which occurs in 5–10% of the asthmatic population. Severe asthma is considered if the patient remains uncontrolled despite optimal treatment. Although, it is only a few percent within the asthmatic population, the care of these patients accounts for more than 60% of the total financial expenditure on asthma (Al-Hajjaj, 2011; Hekking et al., 2015; Highley et al., 2019).

Recently, in a genome-wide association study of asthma in American populations of European origin, the strongest signals were identified at the 9p21.2 locus spanning three genes (*EQTN, MOB3B, TEK*) and consisting of four single nucleotide polymorphisms (SNPs) in strong linkage disequilibrium (LD) with each other. Based on its function, it has been hypothesized that variations in the *TEK* gene were responsible for the association with asthma (Almoguera et al., 2017).

It is well-known that allergic asthma often occurs together with other atopic diseases. Allergic rhinitis and conjunctivitis are the most frequent asthma-associated atopic comorbidities. According to different studies, 50–65% of patients with rhinitis also had conjunctivitis, although conjunctivitis could also exist without rhinitis. Conjunctivitis is an inflammation of the eye characterized by itching, flushing, swelling and the watering of the eyes. Although allergic diseases have genetic backgrounds, there is very little information about allergic conjunctivitis from that point of view (Rosario and Bielory, 2011; Shaker and Salcone, 2016; Fodor et al., 2018). Despite the fact that different kinds of anti-allergic medication used in ophthalmology, patients with allergic conjunctivitis are generally undertreated. According to a French survey, more than 20% of the patients with allergic conjunctivitis have persistent symptoms despite the treatment. Furthermore, symptoms are underestimated because the huge negative effect on the quality of life is frequently ignored and it is misdiagnosed or under-diagnosed due to other disorders of the eye (Klossek et al., 2012). These imply that novel therapeutic targets are needed in both severe asthma and conjunctivitis.

Earlier, we investigated in a pediatric European population whether three expression QTL SNPs in the *TEK* gene influenced the susceptibility to asthma or associated phenotypes. We did not find any significant connection between these SNPs and asthma, however, one of the variations showed a rather strong association with allergic conjunctivitis (Fodor et al., 2018).

In the present study, we aimed to further investigate the possible role of the *TEK* gene in asthma and allergic conjunctivitis involving novel populations. We screened an allergic population (discovery cohort) with 112 SNPs in the *TEK* gene and searched for associations with asthma and allergic conjunctivitis. We selected the top associated SNPs and carried out association studies on different populations (replication cohorts). We also studied the Tie2 related pathways on a mouse model of asthma.

## Materials and Methods

### Study Populations

We performed our study in two stages. The discovery cohort comprised participants from the DesensIT project which is described in a paper of Molnár et al. (2017). Participants were selected from patients of five Hungarian allergic outpatient centers with documented ragweed allergy and/or with clinical history for at least 2 years with peak symptoms in August–September and agreed to participate in the study. Those patients were preferred who had moderate-severe seasonal allergic rhinitis based on Allergic Rhinitis and its Impact on Asthma (ARIA) criteria and whose respiratory symptoms remain troublesome despite avoidance or adequate pharmacologic therapy and interfering with usual daily activities or with sleep during the ragweed pollen season. Only adult and adolescent patients at the age of 14–65 years were included in this population. The patients completed questionnaires, including RQLQ (Rhinoconjunctivitis Quality of Life Questionnaire) and RTSS (rhinoconjunctivitis total symptom score), then lung function tests, physical examination and discussion of the allergy-related status were carried out. A diagnosis of ragweed allergy was made in subjects with positive results of allergen-specific serum IgE and skin prick tests (SPTs) in presence of a positive clinical history for allergic respiratory symptoms with a peak in the August–September months. As these patients during the DesensIT project were treated between 2012 and 2014 with sublingual immunotherapy against ragweed allergy, the allergic subphenotypes which are presented in **Table 1** were determined before the immunotherapy was started. The conjunctivitis was diagnosed by physicians according to the RQLQ and RTSS subscale, drug-usage and physical investigation.

**Table 1:** Characteristics of the stage I discovery cohort.

	Demographic and clinical characteristics	All
	Total (n)	306
		
	Male (n) (%)	136 (44.5)
	Female (n) (%)	170 (55.5)
		
	Age (mean (SD) year)	39.2 (12.3)
		
**Comorbidities**	Rhinitis (n (%))	306 (100)
	Asthma (n (%))	68 (22.1)
	Conjunctivitis (n (%))	232 (75.8)
	Atopic dermatitis (n (%))	23 (7.5)
	Food allergy (n (%))	24 (7.8)

The stage II cohorts consisted of 4 cohorts, namely adult and pediatric asthmatics and the corresponding controls. Altogether, they included 1,258 unrelated individuals, out of which 63.9% were children and 36.1% were adults. In the children population, 264 asthmatic samples were collected from different centers in Hungary. Out of the 454 adult samples, 237 were derived from asthmatic patients from the Asthma ambulance of the National Korányi Institute of TB and Pulmonology and the Department of Pulmonology of Semmelweis University. Asthma was diagnosed according to GINA (Global Initiative for Asthma) guidelines, as described earlier (Ungvári et al., 2012b; Ungvári et al., 2012a; Ungvári et al., 2012c). Patients with severe asthma (GINA 4-5) are described in more detail in a recent paper (van Bragt et al., 2019). Allergic conjunctivitis was found in 101 asthmatic patients. The diagnosis of allergic conjunctivitis was made by physician. Due to the low number of adult asthmatics with conjunctivitis, in the case of conjunctivitis, only children were involved in the statistical analysis. FEV1 (forced expiratory volume in 1 second) values were used to estimate the lung function of the asthmatic patients. Allergy status was determined based on the level of allergen-specific serum IgE or a skin prick test. All participants were Hungarian (Caucasian), out of which about 5% were of Gypsy origin based on the Hungarian statistical database. Information of the study population can be found in **Table 2**.

**Table 2:** Characteristics of the stage II replication cohorts.

Clinical and biological characteristics	Pediatric asthmatic patients, n=264	Adult asthmatic patients, n=237	Pediatric control, n=540	Adult control, n=217
Age ± SD	10.6 ± 3.8	50 ± 16.1	8.7 ± 4.0	30.4 ± 12.5
Sex (Male/Female)	170/94	99/138	347/193	102/115
Comorbidity:				
Conjunctivitis, yes(%)/no	93 (35)/171	8 (10)/71		
Rhinitis, yes(%)/no	165(64)/99	42(18)/195		
Rhinoconjunctivitis, yes(%), no	91(34)/173	8(3)/229		
GINA status:				
Number of patients in GINA 1(%)	79 (30)	90(38)		
Number of patients in GINA 2(%)	159 (60)			
Number of patients in GINA 3(%)	25 (10)			
Number of patients in GINA 4-5(%)	0	138 (58)		
FEV1 (%):				
Under 80%	0	161		
Above 80%	11	74		

GINA, Global Initiative for Asthma; FEV1, forced expiratory volume in 1 sec.

The samples of control children were from the Orthopaedic Department of Budai Children’s Hospital and the Urological Department of Heim Pál Children’s Hospital. The controls showed no symptoms of asthma or allergy, nor any need for medication. The adult controls were all healthy blood donors (**Table 2**).

Written informed consent was provided by all patients or their parent/guardian. A detailed description of patients can be found in **Tables 1** and **2**. The study was carried out according to the Declaration of Helsinki and was approved by the Ethics Committee of the Hungarian Medical Research Council.

### Genotyping

Genomic DNA was isolated from whole blood samples of 1,564 individuals using the QIAamp DNA Blood Midi kit (Qiagen, Maryland, USA) or the iPrep PureLink gDNA Blood kit with iPrep Purification Instrument (Invitrogen, Carslbad, CA, USA).

Participants of the DesensIT project (Molnár et al., 2017) were genotyped by the Genome-Wide Human SNP Array 6.0 (Applied Biosystems, Foster City, CA, USA). In the present study, only 112 SNPs in the TEK gene were evaluated. All genotyped SNPs with >80% call rate located in the genomic region ± 6 kb of the TEK gene were selected and filtered according to the Hardy-Weinberg equilibrium criteria (p-value of chi-square goodness-of-fit test > 0.01) and minimal allele frequency threshold (>0.02). From the initial number of 112 genotyped SNPs, 104 SNPs fulfilled all selection criteria. SNP imputation was not performed.

The KBiosciences Competitive Allele-Specific PCR (KASP) version 4.0 genotyping system was utilized (LGC Genomics, Berlin, Germany) along with the ABI 7900HT Fast Real-Time PCR system (Applied Biosystems, Foster City, CA, USA) to genotype the rs3739542, rs994934, rs622232, rs638203, rs7034505 SNPs. The sixth SNP (rs3824410) was genotyped with TaqMan SNP Genotyping Assay and Master Mix (Applied Biosystems). The TaqMan genotyping system is based on the same method but a different design as the KASP system.

### Gene Expression Measurement in a Mouse Model of Asthma

The animal model and the gene expression measurements are described in detail in an earlier paper (Tölgyesi et al., 2009). Briefly, in this experiment 6- to 8-week-old, female, pathogen-free BALB/c mice were used. Three groups of mice (6 mice/group) were sensitized by intraperitoneal injection of 20 mg Ovalbumin (OVA) emulsified in 2.25 mg aluminium hydroxide (Imject Alum) in a total volume of 100 mL phosphate-buffered saline (PBS) on days 0 and 14. Non-sensitized animals (controls) received Alum combinated with PBS only. On days 28, 29, and 30, mice were challenged with 1% OVA aerosol (in PBS) or PBS only (controls) for 20 min by an ultrasonic nebulizer (Omron, Budapest, Hungary) in a plastic chamber. Measurements of lung resistance were carried out on day 31, 24 h after the last challenge. On days 28 and 30, 4 h after the first and third allergen challenge, respectively, mice in groups 1 and 2 were anaesthetized by an intraperitoneal injection of ketamine and xylazine and the lungs were removed for additional analysis. On day 31, 24 h after the third (last) allergen challenge mice in group 3 and the controls were anaesthetized and airway hyperresponsiveness (AHR) was assessed and then bronchoalveolar lavage fluid (BALF) sampling and lung tissue collections were performed, the identical way as it was performed in groups 1 and 2.

RNA from mouse lung was isolated by RNeasy columns (Qiagen, Valencia, CA, USA). Agilent Whole Mouse Genome Oligo Microarray 4x44K chips were used to analyze gene expression patterns in the lung. The results were deposited in the GEO database with a record number of GSE11911. (GSE11911 - GEO DataSets - NCBI).

### Statistics

Hardy-Weinberg equilibrium (HWE) was tested using the chi-square goodness-of-fit test implemented in the online DeFinetti HWE application (Helmholtz Zentrum München, Institut für Humangenetik, http://ihg.gsf.de/cgi-bin/hw/hwa1.pl) with a significance level of 0.01. SNP data were analyzed using SPSS version 19 (SPSS Inc., Chicago, IL, USA) software. Logistic regression analyses were used to evaluate the association between TEK genotypes and asthma or allergic conjunctivitis. In all multivariate logistic regression analyses, age and gender were included as covariates.

Linkage disequilibrium calculations and haplotype analyses were carried out by Haploview version 4.2 software (Broad Institute, Cambridge, Maine, USA). Single locus and multi-marker haplotype association tests were performed with Pearson’s chi-squared test.

All SNPs that nominally significantly associated with asthma or allergic conjunctivitis in the discovery cohort were selected for further investigation in the stage II cohorts.

Microarray raw data were processed by the Feature Extraction software version 7.5 with default parameter settings for two-color oligonucleotide microarrays and then transferred to GeneSpring 9.02 program (Agilent Technologies, Palo Alto, CA, USA) for further processing. In GeneSpring, the normalization and data transformation steps recommended by Agilent Technologies for two-color data were applied. Statistical analysis was performed using the R statistical software (version 3.5.1) (R Core Team, 2018). T-test was applied to identify genes that were differentially expressed between the respective groups against the common reference control. Log2 transformed fold changes (logFC) between the respective groups and the common reference were calculated for 10 selected genes, namely for Angpt1; Angpt2; Angpt4; Tek; Nfkb1; Kdr (gene for Vegfr2); Pik3ca; Akt1; Nos3; Ptprb (gene for Ve-tpt). The false discovery rate (FDR) multiple testing correction method according to Benjamini and Hochberg was applied with p < 0.05 cutoff in our statistical tests.

## Results

### Stage I SNP Association Studies in Patients With Ragweed Allergy

In our stage I study, we utilized the results of a genome-wide SNP genotyping in a population with documented ragweed allergy with moderate-severe rhinitis (Molnár et al., 2017). Some characteristics of this group are shown in **Table 1**. Here, we evaluated the genotyping results of 112 SNPs in the *TEK* gene. The genotyping and the HWE results, the call rates and information about the SNPs are presented in **Supplementary Table 1**. All the genotype distributions of the SNPs corresponded to the HWE. In our evaluations, we considered only SNPs with a call rate above 80% and a minimal allele frequency > 0.02. In this way, altogether 104 SNPs in the *TEK* gene were evaluated.

In this group, 75.8% of the patients had conjunctivitis and 22.1% asthma. First, we compared the allele and genotype distributions between patients with and without conjunctivitis. The results of the statistical evaluations of the comparisons of allelic frequencies are presented in **Supplementary Table 2** (sheet: singlemarker). In the comparison of allelic frequencies between ragweed allergic patients with and without conjunctivitis, altogether 6 SNPs reached the uncorrected significance level (p < 0.05). The lowest p-value was 0.0048 in the case of rs7034505.

Based on the genotyping results, the linkage disequilibrium map of the *TEK* gene was calculated (**Supplementary Figure 1**). Altogether, 17 haplotypes were identified and their possible associations with conjunctivitis were investigated. The results of the statistical evaluation are presented in **Supplementary Table 2** (sheet: haplotypeblocks). No haplotype showed association with conjunctivitis.

The genotypic tests were carried out using recessive (R; 22 vs 12 + 11 genotypes, where two stands for the risk allele), dominant (D; 22 + 12 vs 11) and additive (A; 11 vs 12 vs 22) models. The results of these tests are shown in **Supplementary Table 3**. In this case, 15 models reached the uncorrected significance level and the strongest association was found for rs638203 in the recessive model (p= 0.006; OR = 7.8 (95% CI: 1.80–33.77)).

According to the results, five SNPs (rs3739542; rs994934; rs622232; rs638203; rs7034505) were selected for further investigations in additional populations.

The same tests were carried out for asthma. The results are shown in **Supplementary Table 4**. In this case, one SNP (rs3824410) reached the uncorrected significance level in three different models, namely in additive (p = 0.0077; OR = 0.49 (95% CI: 0.29–0.83)), dominant (p= 0.02; OR = 0.51 (95% CI: 0.29–0.90)) and also the allelic frequencies were significantly different between the asthmatic and non-asthmatic populations (p = 0.008; OR = 0.51 (95% CI: 0.30–0.84)). According to these results, the rare (T) allele of this SNP in this allergic population might reduce the risk of asthma. This SNP was also selected for further association analysis in stage II study.

### Stage II SNP Association Studies in Adult and Pediatric Asthmatic and Control Patients

The selected six SNPs were genotyped in populations, as presented in **Table 2**. The results of the genotyping are shown in **Supplementary Table 5**. First, we tested whether the selected SNPs were associated with asthma in each age group or with conjunctivitis in children because there were only eight adult asthmatic patients with this symptom thus, they were excluded from this analysis. As can be seen in **Supplementary Table 5**, no allele associated directly with these diseases.

When we investigated the asthmatic children only, the minor allele of the rs622232 SNP was associated with conjunctivitis (p = 0.027; OR = 1.65 (95%CI: 1.06-2.57)). When we examined the two genders separately, however, the rs622232 SNP was associated with conjunctivitis only in boys in both allelic and dominant ways (p=0.004; OR =4.76 (95%CI: 1.54–14.75)), while the minor allele of the SNP rs7034505 was associated with conjunctivitis only in girls (p=0.012; OR=2.42 (95%CI: 1.20–4.90)). The minor allele homozygotes of this SNP showed an increased risk with a high odds ratio to conjunctivitis in girls comparing it to the major allele homozygotes (p=0.019; OR=5.46 (95%CI: 1.24–24.09)) (**Table 3**).

**Table 3:** Selected results and statistical evaluations of the association studies with allergic conjunctivitis in the stage II cohorts.

Phenotype		SNP	Phenotype	MAF	Genotype 11	Genotype 12	Genotype 22	Differences between allelic frequencies		Recessive model 11 + 12 vs. 22		Dominant model 11 vs. 12 + 22		11 vs. 22	
					n(%)	n(%)	n(%)	p	OR(95% CI)	p	OR(95% CI)	p	OR(95% CI)	p	OR(95% CI)
Allergic conjunctivitis	Male	rs622232	Present	0.57	4 (12)	21 (62)	9 (26)	**0.024**	1.92 (1.09–3.04)	0.533	1.34 (0.53–3.37)	**0.004**	4.76 (1.54–14.75)	**0.027**	4.13 (1.11–15.29)
			Asthma without conjunctivitis	0.41	33 (39)	34 (40)	18 (21)								
	Female	rs7034505	Present	0.67	3 (12)	11 (42)	12 (46)	**0.012**	2.43 (1.20–4.90)	0.034	2.96 (1.06–8.12)	0.066	3.38 (0.88–13.02)	**0.019**	5.46 (1.24–24.10)
			Asthma without conjunctivitis	0.46	15 (31)	21 (47)	11 (22)								

MAF, minor allele frequency; Genotype 11, homozygotes for common alleles; Genotype 12, heterozygotes; Genotype 22, homozygotes for the risk alleles; OR, odds ratio; CI, confidence interval; Nominally significant associations are given by bold values.

Within the adult asthmatic group, the rare allele of rs3824410 was negatively associated with severe asthma (p=0.009; OR=0.48 (95% CI: 0.27–0.84)) and similar association was found in the dominant model (p=0.022; OR = 0.45 (95%CI: 0.22–0.90)). When only the adult allergic asthmatic patients were considered, the minor T allele of the rs3824410 SNP also associated with a lower risk of severe asthma (p=0.026; OR=0.39; (95%CI: 0.17–0.91) and p=0.03; OR=0.33; (95%CI: 0.12–0.91)) on the allelic level and in the dominant model, respectively. The above detailed results are summarized in **Table 4**.

**Table 4:** Results of the association tests within the asthmatic group in stage II cohorts.

Phenotype		SNP	Phenotype	MAF	Genotype 11	Genotype 12	Genotype 22	Differences between allelic frequencies		Recessive model 11 + 12 vs. 22		Dominant model 11 vs. 12 + 22		11 vs. 22	
					n(%)	n(%)	n(%)	p	OR(95% CI)	p	OR(95% CI)	p	OR(95% CI)	p	OR(95% CI)
Adult asthma	Allergic and non-allergic	rs3824410	Severe	0.16	70 (71)	26 (26)	3 (3)	**0.009**	0.48 (0.27–0.84)	0.086	0.29 (0.07–1.28)	**0.022**	0.45 (0.22–0.90)	**0.041**	0.23 (0.05–1.04)
			Mild	0.29	27 (52)	20 (38)	5 (10)								
	Allergic	rs3824410	Severe	0.15	22 (73)	7 (23)	1 (4)	**0.026**	0.39 (0.17–0.91)	0.283	0.31 (0.03–2.93)	**0.03**	0.33 (0.12–0.91)	0.155	0.22 (0.02–2.10)
			Mild	0.31	19 (48)	17 (42)	4 (10)								

MAF, minor allele frequency; Genotype 11, homozygotes for common alleles; Genotype 12, heterozygotes; Genotype 22, homozygotes for the risk alleles; OR, odds ratio; CI, confidence interval; Nominally significant associations are given by bold values.

We computed linkage disequilibrium between the six selected SNPs, and these showed no LD (the maximal r-square value was 0.11 between any pairs of SNPs). Therefore, we did not perform a haplotype analysis of these SNPs.

### Investigation of the Possible Role of the Angiopoietin-Tie2 Pathway in a Mouse Model of Asthma

The detailed description of the lung characteristics of mice in the different groups can be seen in an earlier paper (Tölgyesi et al., 2009). Briefly, after intraperitoneal sensitization with OVA on days 0 and 14, the first airway inhalation of allergen on day 28 led to a remarkable neutrophil and macrophage recruitment in BALF samples of the OVA-sensitized mice (group 1). This initial neutrophil inflammation weakened during the progression and almost entirely vanished by the end of the protocol (group 3) while the asthma-related Th2-type eosinophilic airway inflammation and hyperresponsiveness evolved. On day 31 in mice of group 3, the lung resistance measurements revealed strong AHR provoked by methacholine in the OVA-challenged mice compared to controls, demonstrating the development of AHR in mice undergoing OVA sensitization and challenge.

**Figure 2:**
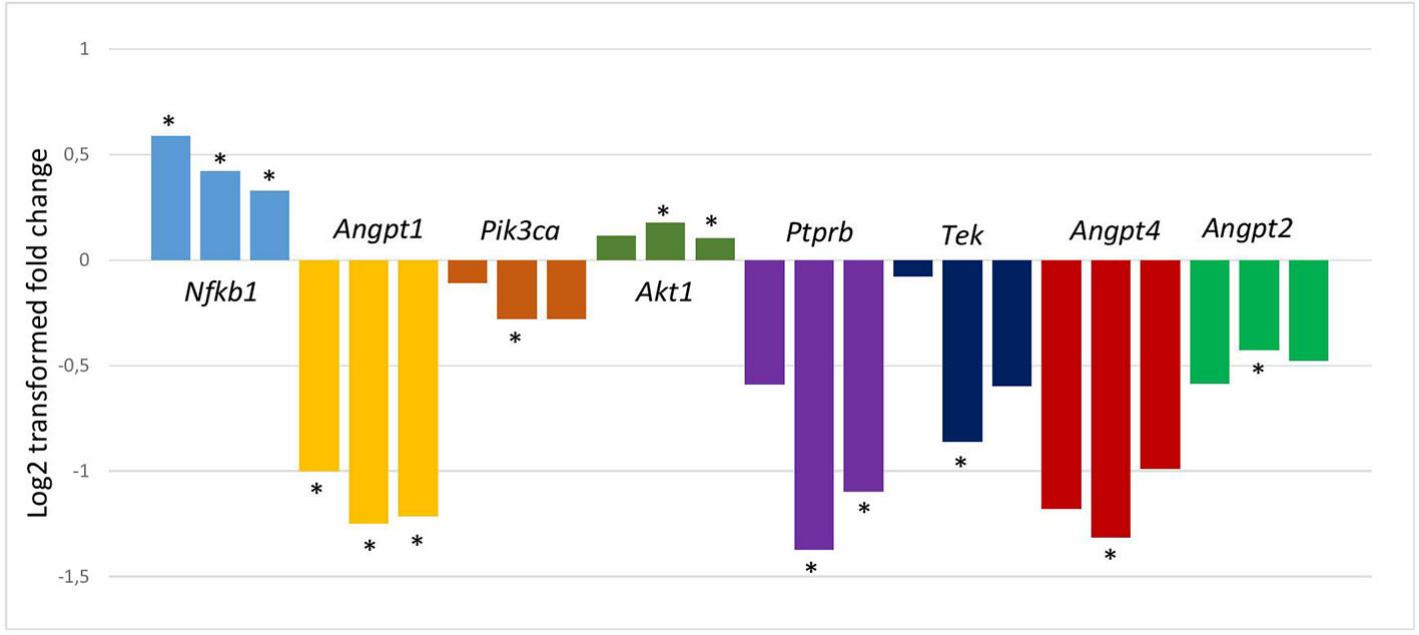
LogFC values of the changes in gene expressions relative to controls. Only those genes are depicted whose expression showed at least one significant change in their expressions during the process leading to Th2-type allergic airway inflammation and hyperresponsiveness in a mouse model of asthma. After intraperitoneal sensitization with OVA on day 0 and 14 on days 28, 29, 30, and 31 mice were placed in a plastic chamber and challenged with OVA aerosol (in PBS) or PBS only (controls) for 20 min by an ultrasonic nebulizer. On day 28, 30, and 31, 4 h after the allergen challenge, in groups 1, 2, and 3, respectively, mice were anaesthetized, and the lungs were removed for gene expression analysis. The measurements were carried out by Agilent Whole Mouse Genome Oligo Microarray 4x44K chips. The results were deposited in GEO database with a record number of GSE11911 (GSE11911 - GEO DataSets - NCBI). *Indicates significant differences.

Microarray analysis of the lung showed a marked alteration in the gene expression profile during the asthmatic response. Out of the 44 thousand transcripts 533, 1,554, and 1,134 transcripts showed more than 2.0-fold statistically significant differential expression in groups 1, 2, and 3 relative to the controls, respectively (Tölgyesi et al., 2009). To investigate the possible role of the Angiopoietin-Tie2 pathway in this model the relative expressions of 10 genes from this pathway were studied (**Figure 1**). The genes which were selected from the scientific literatures were the followings: *Angpt1; Angpt2; Angpt4; Tek; Nfkb1; Kdr* (gene for Vegfr2); *Pik3ca; Akt1; Nos3; Ptprb* (gene for Ve-tpt). The expression levels of the two neighboring genes of the *TEK* gene (*Mob3b* and *Eqtn*) were also measured. The logFC values of the gene expressions and the statistical evaluations are presented in **Supplementary Table 6**. Notably, out of the 10 genes, 8 showed significant changes in gene expressions during the process. In **Figure 2**, the logFC values of the genes which showed at least one significant change in the gene expressions during the process are depicted. As can be seen, the expression levels of most of the selected genes were reduced during the process leading to AHR and eosinophil infiltration in the lung, including *Tek* and *Angpt1*. The expression level of the *Tek* gene reduced statistically significantly in group 2, and it remained lower (although in this case, it was statistically not significant), also in group 3. The expressions of the angiopoietin I gene were significantly lower in all groups. In contrast, the expression of the proinflammatory *Nfkb1* whose expression is negatively regulated by Tie2 and Ang1 was significantly elevated at all points in time. In group 3, the expression level of *Mob3b* showed a slight increase (**Supplementary Table 6**). No other differences were found in case of *Mob3b* and *Eqtn*.”

## Discussion

In the present study, we investigated whether genetic variations in the *TEK* gene influence the susceptibility to asthma or allergic conjunctivitis in multiple populations. We also studied the possible role of Tie2 related pathways on a mouse model of asthma.

The role of variations in the *TEK* gene in human asthma was first suggested by a meta-analysis of genome-wide association studies, that identified four SNPs on chromosome 9p21.2 associated with asthma in a European American population. Although the detected SNPs were quite far from the *TEK* gene, they were in linkage disequilibrium with a missense variant in the *TEK* gene. However, no expression QTL (eQTL) effects were shown in any of the tissues relevant to asthma during the fine mapping of the region (Almoguera et al., 2017). Later, an association study investigating three eQTL SNPs in the *TEK* gene with known respiratory disease association found no connection with asthma (Fodor et al., 2018). In the present study, 112 SNPs were screened in the *TEK* gene and the selected SNPs were investigated in other cohorts. The rs3824410 SNP was found to be associated with asthma in a population with moderate and severe rhinitis and with severe asthma in another population involving asthmatic patients. In all cases, the rare allele (T) of the rs3824410 SNP was associated with lower susceptibility to asthma or to severe asthma with similar odds ratios. These findings are in agreement with the previous suggestion that variations in the *TEK* gene might play a role in asthma in populations of European origin.

Presently, it can only be assumed how the rs3824410 SNP might influence the susceptibility to asthma or the severity of the disease. Investigating the possible function of this SNP with the help of databases and bioinformatic tools shows that the rs3824410 is in no close linkage with other frequent variations in European populations, indicating that it might have an independent role. The SNP might have a function because it locates on a DNase hypersensitivity site in the lung, indicating an open chromatin region. In addition, the SNP modifies the binding sites of two transcription factors, namely CTCF and interferon-stimulated gene factor 3 (ISGF-3) (Matys, 2006; Boyle et al., 2012; Machiela and Chanock, 2015). Earlier, both transcription factors have been found implicated in asthma (Duerr et al., 2016; Schmiedel et al., 2016). Although the SNP changes a less conserved nucleotide within the binding motifs (see **Supplementary Figure 2**) it might have some influence on the strength or time of the binding. It must be noted, that several asthma susceptibility loci are known to contain CTCF motifs (Pascoe et al., 2017).

The important role of the *TEK* gene and the Tie2 pathway in asthma is further strengthened by the observation that the expressions of 8 out of 10 selected genes on the Tie2 related pathway differed significantly from those of controls in a mouse model of asthma during certain time points in the process leading to Th2-type allergic airway inflammation and hyperresponsiveness (**Figure 2**). In the majority of the gene expression studies of asthma, the expressions are measured only at one point; in mice usually at the final stage when the airway inflammation and AHR are already developed or in humans when the patients experience no exacerbation. In the present study, we measured the gene expression changes during the process starting from the first airway inhalation of allergen until the development of the Th2-type allergic airway inflammation at three time points. Only the expression of *Nfkb1* was significantly elevated in all three occasions, which is not surprising, since *Nfkb1* codes the DNA binding subunit of the NFκB transcription factor, which is a hub protein controlling a variety of processes, including inflammation in the asthmatic lung (Liu et al., 2017). Although, the Tie2 pathway is intensely investigated, the exact role of the different participants of the pathway in different cells, processes and diseases are far from clear (Brindle et al., 2006; Augustin et al., 2009; Karar and Maity, 2011; Thurston and Daly, 2012; Souma et al., 2016; Zhang et al., 2019). The detailed discussion of the significance of the expression changes of the different genes is beyond the scope of this paper. We mention here only decreased expressions of the *Tek* and *Angpt1* genes during the whole process. Tie2 which is coded by the *TEK* gene is a tyrosine kinase receptor playing a central role in vascular stability. Ang1 stimulates the activity of the kinase by binding to Tie2 providing an anti‐inflammatory effect (e.g., inhibiting NFκB1) and vessel stabilization (Saharinen et al., 2017). Lower Tie2, together with the lower Ang1 expression can increase inflammatory processes and weaken the barrier function of the endothelial cells in the lung. Furthermore, the inflammatory response to the inhaled allergen can also cause blood vessels to dilate by becoming more permeable. It can be hypothesized that these processes may contribute to the leakage of the activated inflammatory cells into the surrounding tissues in the lung, causing the characteristic symptoms of the allergic airway inflammation. Our findings correlate with another study which showed that Ang1 expression and Tie2 phosphorylation are reduced in the lung of ovalbumin-treated mice (Simoes et al., 2008). The study also suggested that Ang1 can protect against airway inflammation and hyperresponsiveness in asthma.

In the stage I association study of allergic conjunctivitis, we selected 5 SNP in the *TEK* gene with nominally significant p-values. In conjunctivitis in stage II, we studied only children because there were too few adult patients. Here, we found that the genetic background of allergic conjunctivitis is different in the two genders. The sex differences in allergic conjunctivitis is a well-known phenomenon, especially in children (Chen et al., 2008). This condition typically occurs more frequently in the younger populations, between 4 and 12 years of age and more frequently amongst boys. The rs622232 SNP was associated with conjunctivitis only in boys in both allelic and dominant ways with a relatively high odds ratio ((p = 0.004; OR = 4.76), while the minor allele and the minor allele homozygotes of the SNP rs7034505 were associated with an increased risk to conjunctivitis in girls. Both SNPs are in introns and no functions were attached to them in the public databases. The rs622232 is in LD (r^2^ > 0.8) with rs621786 also without a known function (Machiela and Chanock, 2015). The rs7034505 is in LD with 4 SNPs, one of them (rs13293051) changes the binding site of the transcription factor FOXP1 (**Supplementary Figure 3**), which is a transcriptional repressor and is expressed in the retina (FOXP1 Gene; Machiela and Chanock, 2015). No connection between these SNPs and conjunctivitis was previously described.

It must be noted, however, that there are some weaknesses in the study. We investigated several SNPs in different populations and found SNPs associating with conjunctivitis and asthma, but the associations were only nominally significant. If multiple test corrections had been applied, because of the number of SNPs and the relative few patients, the associations would not have been statistically significant. In contrast, in the gene expression study, the p-values were adjusted, i.e., they were corrected for multiple tests (see **Supplementary Table 6**). We think, however, that the collected proofs that the *TEK* gene and its variations might have a role in asthma are quite convincing. The functions of the Tie2, the results of the gene expression in the animal model and the association studies in independent populations strengthen each other, providing accumulating evidence that *TEK* is a potential gene in asthma pathogenesis. Although the supporting results are fewer in conjunctivitis, there are several independent proofs favoring this hypothesis as well. The gene is highly expressed in the lungs, and it is also expressed in the eye. Mutations in the *TEK* gene are associated with several eye diseases, and Tie2 is a highly investigated target in different conditions such as subretinal and choroidal neovascularization, macular oedema or diabetic retinopathy (Campochiaro, 2015; Campochiaro and Peters, 2016; Tan et al., 2017). Moreover, SNPs in the gene showed suggestive associations with conjunctivitis in different studies with high odds ratios (Fodor et al., 2018). The theoretical mechanism leading to allergic conjunctivitis can be similar as it was described in asthma. Naturally, in both cases, additional studies in cell and tissue cultures, animal models and different human populations are needed for more definitive proofs.

In conclusion, our results provide additional proof that the Tie2 pathway, the *TEK* gene and its variations might have a role in asthma and allergic conjunctivitis. The gene and its associated pathways can be potential therapeutic targets in both diseases.

## Data Availability Statement

The datasets generated for this study can be found in the GSE11911, human data in European Variation Archive: Project accession: PRJEB36264, Analysis accession: Affymetrix: ERZ1284184, Genotyping: ERZ1284183.

## Ethics Statement

Written informed consent was signed by all patients or by their parent/guardian.

## Author Contributions

ZG carried out the laboratory experiments, evaluations, and interpretations of data under the supervision of CS. AG and VM contributed to the analyses of data in both microarray and GWAS databases. BB and SP contributed to the performance of GWAS. AN, AK, MS, ZC, LT, and GG helped with the collection of specimens and phenotyping of the individuals. All authors participated in the evaluation of results and read this manuscript.

## Funding

This study was supported by European regional development fund: GOP 1.1.1–11–2011–0016 and National Research, Development and Innovation Office (NKFIH): K81941, K112872 (CS).

## Conflict of Interest

The authors declare that the research was conducted in the absence of any commercial or financial relationships that could be construed as a potential conflict of interest.

## Supplementary Material

Supplementary Table 1 Genotyping and HWE results, call rates and information about the SNPs genotyped in the stage I study.

Supplementary Table 2 Results of the statistical evaluations of the comparisons of the allelic frequencies in the stage I study comparing patients with or without conjunctivitis. The results are presented in sheet ‘singlemarker’. The SNPs selected for the stage II study are in bold. The results of the statistical evaluation investigating 17 haplotypes are presented in sheet ‘haplotypeblocks’.

Supplementary Table 3 Results of the statistical evaluation of the genotypic tests in the stage I study comparing patients with or without conjunctivitis. The models used in these tests are the followings: Recessive (R; 22 vs 12+11 genotypes, where 2 stands for the risk allele), dominant (D; 22+12 vs 11) and additive (A; 11 vs 12 vs 22).

Supplementary Table 4 Results of the statistical evaluations of the association study carried out in the stage I study comparing patients with or without asthma. The abbreviations used in this table (see also in the legend of **Supplementary Table 3**): A: additive; R: recessive; D: Dominant; AF: allelic frequencies.

Supplementary Table 5 Results of the genotyping and the statistical evaluations of the comparisons of allelic frequencies in the stage II study comparing patients with or without asthma (sheet: ‘Asthma’) and conjunctivitis (sheet: ‘Conjunctivitis’).

Supplementary Table 6 The log2 fold change (logFC) values of the gene expressions of the ten selected genes in the lungs of mice at three time points (group1, 2 and 3, see text) leading to Th2-type allergic airway inflammation relative to controls and the statistical evaluations. The significant adjusted p-values are highlighted. The threshold of significance: p < 0.05.

Supplementary Figure 1 Linkage disequilibrium map of the *TEK* gene based on genotyping of 104 SNPs in the gene.

Supplementary Figure 2 Binding sequence of the ISGF-3 transcription factor (Matys, 2006), the nucleotide which is changed by the rs3824410 SNP is marked.

Supplementary Figure 3 Binding sequence of the FOXP1 transcription factor (Matys, 2006), the nucleotide which is changed by the rs13293051 SNP is marked.

## References

[B1] Al-Hajjaj M. (2011). Difficult-to-treat asthma, is it really difficult. Ann. Thorac. Med. 6, 1–2. 10.4103/1817-1737.74268 21264163 PMC3023863

[B2] Almoguera B. Vazquez L. Mentch F. Connolly J. Pacheco J. A. Sundaresan A. S. (2017). Identification of four novel loci in asthma in european american and african american populations. Am. J. Respir. Crit. Care Med. 195, 456–463. 10.1164/rccm.201604-0861OC 27611488 PMC5378422

[B3] Augustin H. G. Young Koh G. Thurston G. Alitalo K. (2009). Control of vascular morphogenesis and homeostasis through the angiopoietin - Tie system. Nat. Rev. Mol. Cell Biol. 10, 165–177. 10.1038/nrm2639 19234476

[B4] Boyle A. P. Hong E. L. Hariharan M. Cheng Y. Schaub M. A. Kasowski M. (2012). Annotation of functional variation in personal genomes using RegulomeDB. Genome Res. 22, 1790–1797. 10.1101/gr.137323.112 22955989 PMC3431494

[B5] Brindle N. P. J. Saharinen P. Alitalo K. (2006). Signaling and functions of angiopoietin-1 in vascular protection. Circ. Res. 98, 1014–1023. 10.1161/01.RES.0000218275.54089.12 16645151 PMC2270395

[B6] Campochiaro P. A. Peters K. G. (2016). Targeting tie2 for treatment of diabetic retinopathy and diabetic macular edema. Curr. Diab. Rep. 16, 126. 10.1007/s11892-016-0816-5 27778249

[B7] Campochiaro P. A. (2015). Molecular pathogenesis of retinal and choroidal vascular diseases. Prog. Retin. Eye. Res. 49, 67–81. 10.1016/j.preteyeres.2015.06.002 26113211 PMC4651818

[B8] Chen W. Mempel M. Schober W. Behrendt H. Ring J. (2008). Gender difference, sex hormones, and immediate type hypersensitivity reactions. Allergy Eur. J. Allergy Clin. Immunol. 63, 1418–1427. 10.1111/j.1398-9995.2008.01880.x 18925878

[B9] Duerr C. U. Mccarthy C. D. A. Mindt B. C. Rubio M. Meli A. P. Pothlichet J. (2016). Type I interferon restricts type 2 immunopathology through the regulation of group 2 innate lymphoid cells. Nat. Immunol. 17, 65–75. 10.1038/ni.3308 26595887 PMC9135352

[B10] Fodor L. E. Gézsi A. Ungvári I. Semsei Á.F. Gál Z. Nagy A. (2017). Investigation of the possible role of the Hippo/YAP1 pathway in asthma and allergy. Allergy. Asthma. Immunol. Res. 9, 247–256. 10.4168/aair.2017.9.3.247 28293931 PMC5352576

[B11] Fodor L. E. Gézsi A. Gál Z. Nagy A. Kiss A. Bikov A. (2018). Variation in the TEK gene is not associated with asthma but with allergic conjunctivitis. Int. J. Immunogenet. 45, 102–108. 10.1111/iji.12365 29667338

[B12] FOXP1 Gene Available at: https://www.genecards.org/cgi-bin/carddisp.pl?gene=FOXP1.

[B13] GSE11911 - GEO DataSets - NCBI Available at: https://www.ncbi.nlm.nih.gov/gds/?term=GSE11911%5BAccession%5D [Accessed November 18, 2019].

[B14] Hekking P. P. W. Wener R. R. Amelink M. Zwinderman A. H. Bouvy M. L. Bel E. H. (2015). The prevalence of severe refractory asthma. J. Allergy Clin. Immunol. 135, 896–902. 10.1016/j.jaci.2014.08.042 25441637

[B15] Highley A. D. Cookman C. Morrow L. E. Malesker M. A. (2019). Severe asthma: an update for 2019. U.S. Pharm. 44, HS2–HS7. Available at: https://www.medscape.com/viewarticle/917192 [Accessed December 18, 2019].

[B16] Karar J. Maity A. (2011). PI3K/AKT/mTOR pathway in angiogenesis. Front. Mol. Neurosci. 4, 51. 10.3389/fnmol.2011.00051 PMC3228996 22144946

[B17] Klossek J. M. Annesi-Maesano I. Pribil C. Didier A. (2012). The burden associated with ocular symptoms in allergic rhinitis. Int. Arch. Allergy Immunol. 158, 411–417. 10.1159/000334286 22487783

[B18] Liu T. Zhang L. Joo D. Sun S. C. (2017). NF-κB signaling in inflammation. Signal Transduction Targeting Ther. 2, 17023. 10.1038/sigtrans.2017.23 PMC5661633 29158945

[B19] Machiela M. J. Chanock S. J. (2015). LDlink: a web-based application for exploring population-specific haplotype structure and linking correlated alleles of possible functional variants. Bioinformatics 31, 3555–3557. 10.1093/bioinformatics/btv402 26139635 PMC4626747

[B20] Matys V. (2006). TRANSFAC(R) and its module TRANSCompel(R): transcriptional gene regulation in eukaryotes. Nucleic Acids Res. 34, D108–D110. 10.1093/nar/gkj143 16381825 PMC1347505

[B21] Molnár V. Nagy A. Tamási L. Gálffy G. Böcskei R. Bikov A. (2017). From genomes to diaries: A 3-year prospective, real-life study of ragweed-specific sublingual immunotherapy. Immunotherapy 9, 1279–1294. 10.2217/imt-2017-0093 29130793

[B22] Pascoe C. D. Obeidat M. Arsenault B. A. Nie Y. Warner S. Stefanowicz D. (2017). Gene expression analysis in asthma using a targeted multiplex array. BMC Pulm. Med. 17 (1), 189. 10.1186/s12890-017-0545-9 PMC5725935 29228930

[B23] R Core Team (2018). R: A language and environment for statistical computing. https://www.R-project.org/.

[B24] Rosario N. Bielory L. (2011). Epidemiology of allergic conjunctivitis. Curr. Opin. Allergy Clin. Immunol. 11, 471–476. 10.1097/ACI.0b013e32834a9676 21785348

[B25] Saharinen P. Eklund L. Alitalo K. (2017). Therapeutic targeting of the angiopoietin-TIE pathway. Nat. Rev. Drug Discovery 16, 635–661. 10.1038/nrd.2016.278 28529319

[B26] Schmiedel B. J. Seumois G. Samaniego-Castruita D. Cayford J. Schulten V. Chavez L. (2016). 17q21 asthma-risk variants switch CTCF binding and regulate IL-2 production by T cells. Nat. Commun. 7, 13426. 10.1038/ncomms13426 PMC5116091 27848966

[B27] Shaker M. Salcone E. (2016). An update on ocular allergy. Curr. Opin. Allergy Clin. Immunol. 16, 505–510. 10.1097/ACI.0000000000000299 27490123

[B28] Simoes D. C. M. Vassilakopoulos T. Toumpanakis D. Petrochilou K. Roussos C. Papapetropoulos A. (2008). Angiopoietin-1 protects against airway inflammation and hyperreactivity in asthma. Am. J. Respir. Crit. Care Med. 177, 1314–1321. 10.1164/rccm.200708-1141OC 18356565

[B29] Souma T. Tompson S. W. Thomson B. R. Siggs O. M. Kizhatil K. Yamaguchi S. (2016). Angiopoietin receptor TEK mutations underlie primary congenital glaucoma with variable expressivity. J. Clin. Invest. 126, 2575–2587. 10.1172/JCI85830 27270174 PMC4922711

[B30] Tölgyesi G. Molnár V. Semsei Á.F. Kiszel P. Ungvári I. Pócza P. (2009). Gene expression profiling of experimental asthma reveals a possible role of paraoxonase-1 in the disease. Int. Immunol. 21, 967–975. 10.1093/intimm/dxp063 19556304

[B31] Tan G. S. Cheung N. Simó R. Cheung G. C. M. Wong T. Y. (2017). Diabetic macular oedema. Lancet Diabetes Endocrinol. 5, 143–155. 10.1016/S2213-8587(16)30052-3 27496796

[B32] Temesi G. Virág V. Hadadi É. Ungvári I. Fodor L. E. Bikov A. (2014). Novel genes in human asthma based on a mouse model of allergic airway inflammation and human investigations. Allergy. Asthma. Immunol. Res. 6, 496–503. 10.4168/aair.2014.6.6.496 25374748 PMC4214969

[B33] Thurston G. Daly C. (2012). The complex role of angiopoietin-2 in the angiopoietin-tie signaling pathway. Cold Spring Harb. Perspect. Med. 2 (9), a006550. 10.1101/cshperspect.a006650 22951441 PMC3426817

[B34] Ungvári I. Hadadi É. Virág V. Bikov A. Nagy A. Semsei Á.F. (2012a). Implication of BIRC5 in asthma pathogenesis. Int. Immunol. 24, 293–301. 10.1093/intimm/dxs007 22336533

[B35] Ungvári I. Hadadi É. Virág V. Nagy A. Kiss A. Kalmár Á. (2012b). Relationship between air pollution, NFE2L2 gene polymorphisms and childhood asthma in a Hungarian population. J. Community Genet. 3, 25–33. 10.1007/s12687-011-0075-8 22207565 PMC3266964

[B36] Ungvári I. Hullám G. Antal P. Kiszel P. S. Gézsi A. Hadadi É. (2012c). Evaluation of a partial genome screening of two asthma susceptibility regions using bayesian network based bayesian multilevel analysis of relevance. PloS One 7 (3), e33573. 10.1371/journal.pone.0033573 22432035 PMC3303848

[B37] van Bragt J. J. M. H. Adcock I. M. Bel E. H. D. Braunstahl G.-J. Ten Brinke A. Busby J. (2019). Characteristics and treatment regimens across ERS SHARP severe asthma registries. Eur. Respir. J., 55 (1), 1901163. 10.1183/13993003.01163-2019 31601713

[B38] Zhang Y. Kontos C. D. Annex B. H. Popel A. S. (2019). Angiopoietin-tie signaling pathway in endothelial cells: a computational model. iScience 20, 497–511. 10.1016/j.isci.2019.10.006 31655061 PMC6806670

