# Corrigendum: Investigation of the Possible Role of Tie2 Pathway and *TEK* Gene in Asthma and Allergic Conjunctivitis

**DOI:** 10.3389/fgene.2020.00702

**Published:** 2020-07-10

**Authors:** Zsófia Gál, András Gézsi, Viktor Molnár, Adrienne Nagy, András Kiss, Monika Sultész, Zsuzsanna Csoma, Lilla Tamási, Gabriella Gálffy, Bálint L. Bálint, Szilárd Póliska, Csaba Szalai

**Affiliations:** ^1^Department of Genetics, Cell- and Immunobiology, Semmelweis University, Budapest, Hungary; ^2^MTA-SE Immune-Proteogenomics Extracellular Vesicle Research Group, Semmelweis University, Budapest, Hungary; ^3^Department of Measurement and Information Systems, Budapest University of Technology and Economics, Budapest, Hungary; ^4^Institute of Genomic Medicine and Rare Disorders, Semmelweis University, Budapest, Hungary; ^5^Department of Allergology, Heim Pál Children's Hospital, Budapest, Hungary; ^6^Department of Urology, Heim Pál Children's Hospital, Budapest, Hungary; ^7^Department of Ear, Nose and Throat, Heim Pál Children's Hospital, Budapest, Hungary; ^8^Outpatient Care for Allergy and Asthma, National Korányi Institute of TB and Pulmonology, Budapest, Hungary; ^9^Department of Pulmonology, Semmelweis University, Budapest, Hungary; ^10^Adult Inpatient Care, Pulmonology Hospital Törökbálint, Törökbálint, Hungary; ^11^Department of Biochemistry & Molecular Biology, Genomic Medicine & Bioinformatic Core Facility, University of Debrecen, Debrecen, Hungary; ^12^Department of Research and Development, Heim Pál Children's Hospital, Budapest, Hungary

**Keywords:** asthma, conjunctivitis, Tie2 pathway, *TEK* gene, angiopoietin (Ang)

In the published article, there was an error concerning affiliation of **András Gézsi**. In addition to affiliation **1**, he is also affiliated to:

MTA-SE Immune-Proteogenomics Extracellular Vesicle Research Group, Semmelweis University, Budapest, Hungary

Department of Measurement and Information Systems, Budapest University of Technology and Economics, Budapest, Hungary

The authors apologize for this error and state that this does not change the scientific conclusions of the article in any way. The original article has been updated.

