# Supplementary figures and images for: Investigation of the Possible Role of Tie2 Pathway and TEK Gene in Asthma and Allergic Conjunctivitis
Source: Front Genet. 2020 Feb 27;11:128. doi: 10.3389/fgene.2020.00128 (PMC7057532; doi:10.3389/fgene.2020.00128)

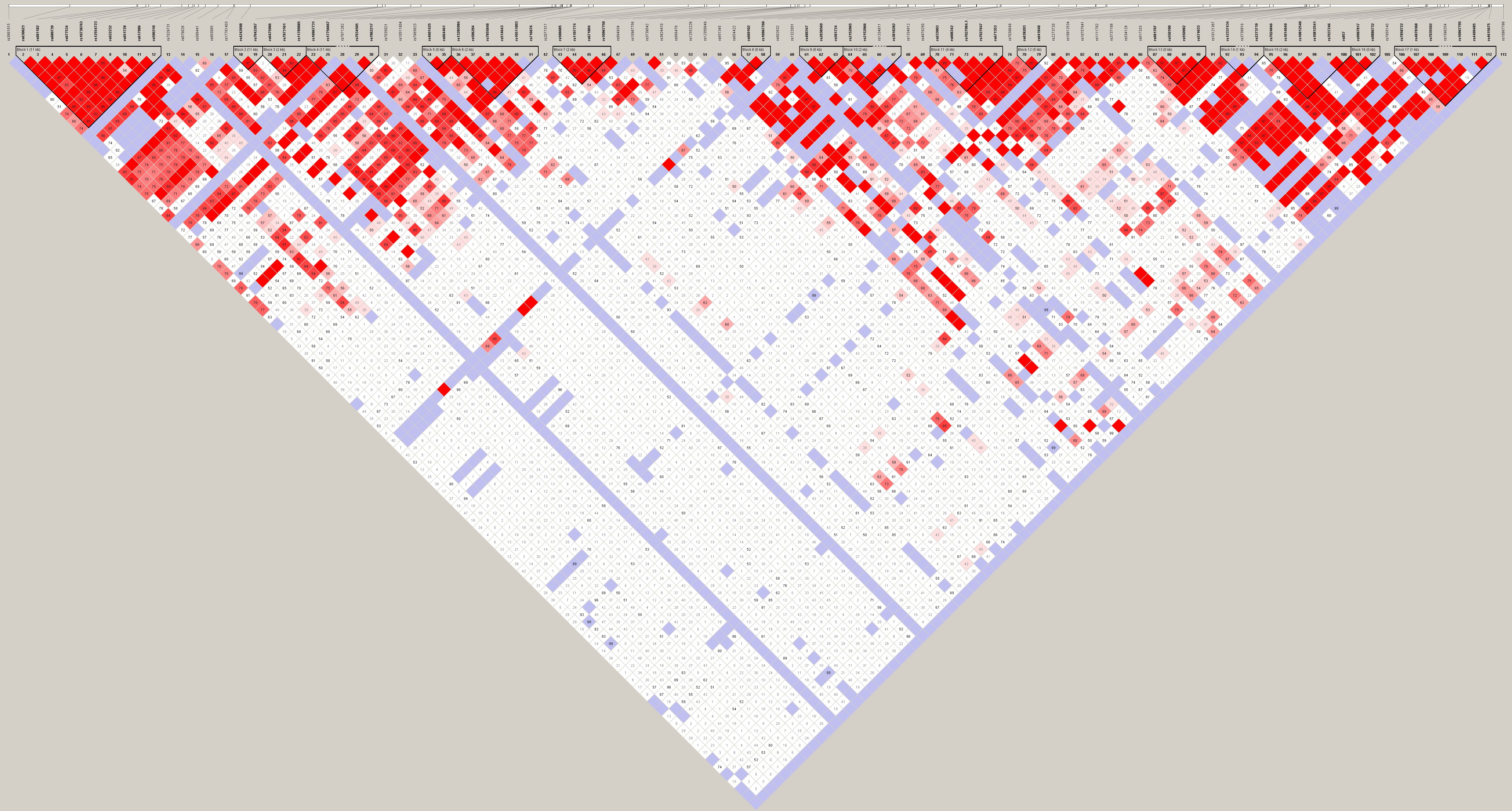

Supplement: Supplementary Figure 1 — Linkage disequilibrium map of the TEK gene based on genotyping of 104 SNPs in the gene. [file Image_1.jpg]

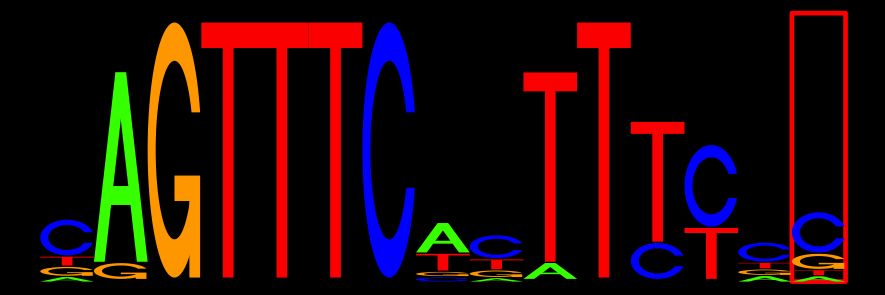

Supplement: Supplementary Figure 2 — Binding sequence of the ISGF-3 transcription factor (Matys, 2006), the nucleotide which is changed by the rs3824410 SNP is marked. [file Image_2.jpg]

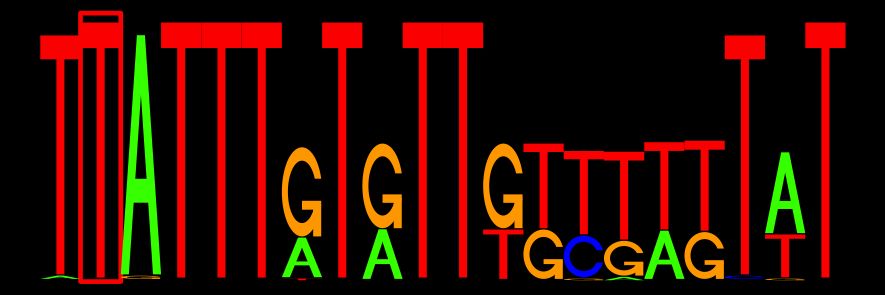

Supplement: Supplementary Figure 3 — Binding sequence of the FOXP1 transcription factor (Matys, 2006), the nucleotide which is changed by the rs13293051 SNP is marked. [file Image_3.jpg]
